# 

**Published:** 1916-02-12

**Authors:** 


### WILL EVERY READER PLEASE HELP?

**In the interests of this country, the Empire, and our Allies the Government have intimated an immediate reduction in the importation of paper, paper pulp, and other materials for paper-making. This necessary action of the Government makes it imperative that not a single copy of "The Hospital" shall be wasted, to which end returns must be stopped. This can only be accomplished by readers of "The Hospital" assisting us to the utmost to help the Government and our Allies, by giving an order to their newsagent or bookseller to-day for their weekly copy of "The Hospital." May we rely upon them thus to join hands with the Editor in helping to shorten the war? Every reader who at once places an order with his newsagent will co-operate to the full in this matter, and will render the completest possible help to our country and our Allies.**

### Leeds General Infirmary.

We have been favoured with an advance copy of the statement of income and expenditure for 1915, and the tables of admissions and attendances of patients for the same period. It is much to the credit of the administration that these figures should so quickly and in so convenient a form be placed in the hands of those who are interested in this great hospital.

A feature in the arrangement of the details which is specially useful is the comparison throughout, as regards income, expenditure, statistics of patients, and weights of provisions used, of the year under review with the previous year. This helpful presentation of essential information might, with profit, be more generally adopted. But at the present moment, when all hospital administrators are naturally curious to examine every important piece of authentic information which will throw a light on the effects of the unparalleled conditions of the last eighteen months upon hospital work, the statistics of the General Infirmary at Leeds are particularly worthy of close examination.

As so often happens in respect of institutional finance, one meets the unexpected. In practically every item of income an increase is shown, and where that barometer of hospital prosperity—the amount of the annual subscription list—might be expected to show a decline we find a marked rise. On the other side it is also satisfactory to note that the ordinary expenditure for 1915 (£41,787) is appreciably below that for 1914 (£43,567), but as this result is mainly accounted for by a decrease of £1,780 under "surgery" and "dispensary," we must look beyond the financial figures for an explanation. The daily average occupation of beds, it is shown, was practically the same in the two years compared, but there has been a large decrease in the amount of work done in the out-patient department. The number of new out-patients fell from 23,153 to 16,054, the casualties from 14,450 to 12,073, and the maternity cases from 248 to 180. The total attendances fell from 176,669 to 123,892—a decrease of 52,777 attendances, which may easily account for the main saving under drugs and dressings after allowing for the substantial rise in prices. No doubt the forthcoming annual report will disclose the reasons explaining these results.

Some of the details of quantities and gross cost of provisions are indicative of the present trials of the commissariat department: 25,000 lb. of fish cost £693, as against £539; 28,000 eggs in 1915 cost £220, while £248 bought 40,000 in 1914; 120,000 lb. of flour cost £935 as against £629.

Five hundred and one British and Belgian soldiers have been treated as in-patients since the commencement of the war.

### Buildings Contemplated.

Leamington.—Alterations and additions will be made to the Warneford and South Warwickshire Hospital at a cost of about £1,200.

Marple.—Plans have been prepared for a sanatorium at Nab Top, Marple.

Penrhiewtyn.—The Neath and District Hospital, situated at Penrhiewtyn, which was built by the Neath Guardians, at a cost of about £36,000, as an infirmary for the Neath Union, has been formally opened. Mr. J. Cook Rees was the architect, and Messrs. Evan Thomas and Sons, of Neath, were the contractors.

### Passing Events and Topics.

### The University of Sheffield.

The Council, at its meeting on February 7, appointed Arthur J. Hall, M.A., M.D., F.R.C.P. (senior physician, Sheffield Royal Hospital), to the Professorship of Medicine, in succession to Dr. Duncan Burgess.

### Medical Men in France and Italy.

Some interesting statistics are to hand concerning the medical profession in France and Italy. In France before the war there were about 20,000 medical men, of whom 3,000 resided in Paris. It is estimated that the income of the majority of the Parisian practitioners was less than £160 a year. There are eighty medical members of the Chamber of Deputies. In Italy there are 22,700 medical men, or about six to every 10,000 of population; in Naples, however, the proportion is twelve to each 10,000 inhabitants, and in Rome there is one doctor to each 10,000 of the population.

### Royal Infirmary, Edinburgh.

NURSES PRIZE-GIVING.

Owing to the war the prize-giving was not held in 1915, but took place quietly this year. At 4 o'clock on Monday, February 7, the managers, some members of the nursing-staff, and the prize-winners assembled in the Recreation-room. The prizes were distributed by Sir Robert K. Inches, Lord Provost of the city, and Mrs. George Kerr, convener of the nursing committee, made a few well-chosen remarks. After the company had partaken of tea the managers adjourned to the usual meeting of the board. The list of awards is as follows:—

1914.—**Hygiene:** First prize (equal), Nurse R. Grant and Nurse J. Grey. **Gynæcology:** First prize (equal), Nurse Jamieson and Nurse M. C. Thomson; second prize, Nurse Nicholson. **Medical Nursing:** First prize, Nurse Alston; second prize, Nurse A. Taylor. **Surgical Nursing:** First prize, Nurse E. Thomson; second prize, Nurse Hopekirk. **Bandaging:** First prize, Nurse Christie; second prize, Nurse Stables.

1915.—**Anatomy and Physiology:** First prize, Nurse E. Thomson; second prize, Nurse E. Forsyth. **Materia Medica:** First prize, Nurse Alston; second prize (equal), Nurse Pollock and Nurse Macdougall. **Gynæcology:** First prize, Nurse R. Grant; second prize, Nurse J. Waters. **Hygiene:** First prize, Nurse Green; second prize, Nurse R. Morrison.

### Leicester's Magnificent Red Cross Effort.

The Mayor and Mayoress of Leicester (Alderman and Mrs. North) may be said to have achieved the great success of their two-year mayoralty when they took upon themselves the appeal for funds on behalf of the British Red Cross Society and the Order of St. John of Jerusalem. At an enthusiastic public meeting, Alderman Tollington (Hon. Treasurer of the Fund) reported that a grand total of £12,000 had been collected for an expenditure of under £100. Of this sum £6,481 was contributed by the Leicester hosiery manufacturers, £1,350 by Leicester spinners, £738 by Leicester dyers, and £734 by the secondary, elementary, and private school children of the borough. The Mayor expressed devout thankfulness for the magnificent result, which had brought Leicester into a very prominent position among the towns of the United Kingdom as a contributor to two invaluable Associations. He acknowledged the great help of Mr. E. C. Kemp, the Hon. Organising Secretary, and of the committee and ladies of the Alexandra Day Organisation. The effort of the school-children was one which he felt specially pleased with, and he acknowledged the interest of the teachers in securing the result. Great enthusiasm prevailed when Mr. Henry Howe (President of the Midland Counties Hosiery Association) handed the Mayor a cheque for £6,481 as the result of the local effort among the hosiery manufacturers, a result which they were all very proud of and which was largely due to the energy of Mr. B. W. Russell, the Hon. Treasurer. A musical evening concluded a remarkable meeting.

Colonel A. D. Sharp, C.M.G., F.R.C.S., A.M.S., honorary surgeon for ear, nose, and throat diseases to the Leeds Public Dispensary, and honorary laryngologist to the Leeds Tuberculosis Association, has been appointed Assistant Director of Medical Services to the 49th West Riding Division in France.

Rates of Subscription: Twelve months, 6/6; Six months, 4/-; Three months, 2/-, post free to any address in the United Kingdom. Abroad, Twelve months, 8/8; Six months, 5/-; Three months, 2/6, post free.—Address The Subscription Dept., "The Hospital," The Hospital Building, 28/29 Southampton Street, Strand, London, W.C.

